# Learning by doing and creation of the shared discovery curriculum

**DOI:** 10.1080/10872981.2023.2181745

**Published:** 2023-02-25

**Authors:** Aron Sousa, Brian Mavis, Heather Laird-Fick, Robin DeMuth, Jonathan Gold, Matthew Emery, Gary Ferenchick, Anthony Paganini, Migdalisel Colon-Berlingeri, Cindy Arvidson, Helga Toriello, Carol Parker, Robert Malinowski, Churlsun Han, Dianne Wagner

**Affiliations:** a Medicine and dean, Michigan State University College of Human Medicine, East Lansing, MI, USA; b Office of Medical Education Research and Development, Michigan State University College of Human Medicine, East Lansing, MI, USA; c Medicine and director of assessment, Michigan State University College of Human Medicine, East Lansing, MI, USA; d Family medicine and associate dean for undergraduate medical education, Michigan State University College of Human Medicine, East Lansing, MI, USA; e Pediatrics and human development, and director of learning societies, Michigan State University College of Human Medicine, East Lansing, MI, USA; f Emergency medicine and director of simulation, Michigan State University College of Human Medicine, Grand Rapids, MI, USA; g Medicine and director of just in time/chief complaints and concerns, Michigan State University College of Human Medicine, East Lansing, MI, USA; h Physiology and director of innovation and integration, Michigan State University College of Human Medicine, East Lansing, MI, USA; i Physiology and director of early clinical experience, Michigan State University College of Human Medicine, Grand Rapids, MI, USA; j Microbiology and molecular genetics, and director of middle clinical experience, Michigan State University College of Human Medicine, East Lansing, MI, USA; k Intersessions, Michigan State University College of Human Medicine, Grand Rapids, MI, USA; l Dean for program evaluation and continuous quality improvement, Michigan State University College of Human Medicine, East Lansing, MI, USA; m The Office of Medical Education Research and Development and associate director of assessment, Michigan State University College of Human Medicine, East Lansing, MI, USA; n Medicine, Michigan State University College of Human Medicine, East Lansing, MI, USA; o Emeritus of medicine, Michigan State University College of Human Medicine, East Lansing, MI, USA

**Keywords:** Competency-based education, undergraduate medical education, curriculum, curricular redesign, learning objectives

## Abstract

**Background:**

The Michigan State College of Human Medicine began as an experiment to teach medical students in community-based settings and to create a primary care workforce for the state. Decades later, CHM faced internal and external challenges that spurred creation of a new curriculum – the Share Discovery Curriculum – founded on learning by doing and other learning theories.

**Methods:**

A curricular design group (CDG) developed guiding principles for reform. Based on this, pedagogies and structures were selected to achieve this vision and developed into a curricular structure. Components of the first-year curriculum were piloted with a group of students and faculty members.

**Results:**

Six guiding principles were endorsed, grounded in learning theories such as Dewey’s Learning by Doing. Based upon these, several key features of the new curriculum emerged: learning communities; one-on-one coaches for students; symptom-based presentations for content; simulation, authentic clinical tasks, flipped classrooms, and modified practice-based learning as primary teaching modalities; early, integrated clinical and scientific learning; milestones as course learning objectives; and a multidimensional, competency-based assessment system.

**Discussion:**

The process and outcomes described here are intended as an exemplar for schools undertaking curricular change. Early stakeholder engagement, faculty development, sustainable administrative systems, and managing complexity are core to the success of such endeavors.

## Introduction

Michigan State University College of Human Medicine (CHM) was created as an experiment in a time of social and educational change, in a new model intended to address physician workforce shortages in the state. To accomplish this mission, CHM immersed students in communities, urban to rural, with clerkship placements across the state. In 1990, the preclinical curriculum was further modified to 1 year of traditional, disciplinary coursework and a second year of problem-based learning (PBL) to enhance students’ ability to apply scientific knowledge to clinical scenarios [1]. In the ensuing years, however, dramatic changes in technological capabilities and increasing pressure to ‘ace the boards’ changed the dynamic within lecture halls and classrooms. By 2010, CHM faced an array of internal and external challenges, including the Carnegie Foundation for the Advancement of Teaching report [2] calling for change (Table 1).

**Table 1.** Challenges driving curricular reform at the college of human medicine.

Internal challenges	External challenges
Falling class attendance as lectures were increasingly broadcast and/or recorded	Carnegie Foundation call for change to focus on learning outcomes, integrated learning, innovation, and professional identity formation [1]
Student anxiety about performance on the United States Medical Licensing Examination USMLE) Step 1	Competition for residency training positions, with USMLE Step 1 scores being used in the National Residency Match Program process
Growing demand from students for Step 1-focused content and resistance to content such as clinical skills training not represented heavily on the examination	Increasing accessibility of well-structured and educationally-sound third party content, in turn impacting faculty roles and faculty-student interactions
Sense of separation between students and patient care	Competition for community clinical sites from other medical schools in the state
Stagnating student performance on a summative third-year objective structured clinical examination (OSCE) for consecutive cohorts	
Doubling of class size with establishment of a second 4-year campus, with up to 100 students per campus per year	

Against this backdrop, CHM undertook major curricular reform with the goal of graduating medical students who would be great first-day residents [3]. The endeavor was grounded primarily in Dewey’s theory of learning by doing, which emphasizes the importance of lived experience for knowledge acquisition [4]. This paper describes the process that led to the competency-based Shared Discovery Curriculum.

## Methods

CHM convened a 10-member Curriculum Design Group (CDG) composed of educational leaders and a purposeful sample across the clinical departments to begin the work of curricular change. The group used a PBL exercise to guide its discussion of mission and guiding principles [5]. Faculty members analyzed cases, explored learning issues, and reflected on personally impactful educational experiences. The CDG used group process to reach a consensus on its final recommendations, which were presented to the Curriculum Committee for approval.

Next, the CDG began to design the content and structure of the new curriculum in keeping with the endorsed principles. Members drew upon the Carnegie Foundation report, medical education literature, and learning theory for this work. The CDG templated and created the first Chief Complaints and Concerns (C3) documents, describing the requisite knowledge and skills related to common symptom-based presentations (e.g., chest pain). The topics themselves were adapted from the Medical Council of Canada Qualifying Examination Objectives [6]. These first C3s were used as exemplars for subsequent faculty boot camps during which groups of clinicians, basic scientists, and social scientists defined specific end-competencies and then worked backwards to identify the timing and distribution of content across learning experiences.

Key components of the Year 1 curriculum were pilot tested. Twenty-one students, who had either just completed the M1 year or were poised to matriculate in medical school, were hired to participate. Faculty members were assigned to facilitate groups and simulation experiences, and ambulatory clinics recruited to accept the pilot students. Students spent 3 half-days per week in community clinics and 6 hours in PBL groups with a clinician-educator debriefing their clinical experiences and discussing cases. Students completed an objective structured clinical exercise (OSCE) as an early Progress Clinical Skills Examination [7]. At the end of the pilot, students and faculty members reflected upon their experiences. Feedback was used to further develop curricular content and delivery.

As curricular content and structure formed, the college’s existing competencies (Table 2) were elaborated into milestones to serve as learning objectives for integrated courses, reinforce competency-based outcomes, and align content across the 4-year curriculum [8].

**Table 2.** SCRIPT competencies mapped to ACGME competencies.

**Service/No ACGME-related competency** Participates in provision of beneficial services within the community Demonstrates preparation and planning to provide services that respond to community need Demonstrates reflection of their participation in service activities
**Care of Patients/Patient Care and Interpersonal Communication Skills** Demonstrates kindness and compassion to patients and their families Collects complete and accurate patient data Synthesizes patient and laboratory data to formulate reasonable assessments and plans Incorporates patient values into illness assessment and care plans Communicates effectively in writing and orally Effectively counsels and educates patients and their families
**Rationality/Practice-Based Learning and Improvement** Identifies personal strength and weaknesses and develops ongoing personal learning plan Demonstrates receptiveness to faculty and peer/colleague feedback as a means of facilitating personal and professional improvement Locates, appraises, and assimilates evidence from scientific studies related to their patients’ health problems
**Integration/Systems-Based Practice** Demonstrates awareness of cost and access issues in the formulation of patient care plans Demonstrates respect for all members of the health-care team Demonstrates understanding of and contributes to a culture of safety Demonstrates knowledge of differing types of medical practice and delivery systems and their implications for controlling health-care allocation and cost Demonstrates knowledge of how social and economic systems in which people live impact health, delivery of health care, and well being
**Professionalism/Professionalism** Demonstrates receptiveness to feedback from faculty/peers/colleagues/team members Contributes actively to group/team process Demonstrates respect to patients, colleagues, and team members Fulfills responsibilities in courses and clinical rotations Takes responsibility for patient outcomes and is accountable to the team, the system of delivery, the patient, and the greater public
**Transformation/Medical Knowledge** Applies essential basic, social, clinical science, and systems knowledge in the care of patients Creates new knowledge through research Participates in lifelong teaching and learning with peers, trainees, and patients

## Results

The Curriculum Committee endorsed the CDG’s six organizing principles (Table 3). The principles, rooted in learning theories, were then used to direct curricular pedagogy and structures (Table 3).

**Table 3.** Principles and learning theories used to design curriculum.

Principle	Associated learning theory	Curricular pedagogy or structure
Maximize student time spent with patients, faculty members, or both	Situated learning theory	Adopt learning society structure to link faculty fellows and students across the curriculum Support continuity for preceptors and students using scholar groups across first 2 years of curriculum Use formative simulation to learn and apply patient care skills, integrate scientific knowledge Begin students’ authentic clinical experiences within the first semester of the curriculum
Support student growth through learning by doing and being useful	Learning as Doing Experiential Learning Theory	Ensure that students are performing authentic clinical tasks, such as performing medication reconciliation or obtaining vital signs in clinics in the first semester, and growing in responsibility thereafter
Use evidence-based approaches to instructional design, instructional strategies, and student achievement		Create learning communities Integrate scientific and clinical learning within courses Introduce flipped classrooms for large and small group activities Use individual and team Readiness Assessment Tests for large group activities Engage in active learning in simulation and laboratories Create an assessment suite with progress testing, multisource feedback, direct observations of clinical skills, and portfolios
Align curriculum and assessment with what matters in the real world	Learning as Doing Adult learning theory Situated learning theory	Create milestones and learning objectives to align curriculum and assessment Align graduation expectations with the Accreditation Council for Graduate Medical Education core competency expectations for entering residents Align progress tests with the USMLE Step examinations and licensing requirements Use symptom-based presentations to structure curricular content Use realistic patient scenarios for objective structured OSCEs Create workplace based assessments modeled on the Association for American Medical College’s Core Entrustable Professional Activities and other authentic physician tasks
Give continual attention to student wellness	Maslow’s hierarchy of needs	Facilitate longitudinal relationships among faculty fellows and students through learning communities Use scholar group fellows as coaches for students, with one-on-one meetings twice per semester Incorporate wellness into competencies and learning objectives Incorporate required wellness activities through Student Affairs
Integrate basic, clinical, and social sciences with patient care	Learning by doing Adult learning theory Situated learning theory	Use modified problem-based learning for small group sessions across first 2 years Link formative simulation activities to symptom-based presentations and underlying scientific principles Assess application of scientific knowledge in OSCE post encounters and link multiple choice question examinations to clinical scenarios Embed learning objectives regarding basic and social sciences within clinical experiences

Learning Societies structure student and faculty interactions. Individuals are assigned to one of the four learning societies, then to a scholar group of one faculty member (also known as a fellow) and eight students. Scholar groups meet 1–2 times weekly in the Early and Middle Clinical Experiences (M1 and M2, respectively) to discuss patient cases and the underlying scientific content. Basic and social scientists rotate through groups to aid in discussions. Scholar groups maintain continuity across the first 2 years of the curriculum and across educational activities such as small group sessions, large group activities with teamwork elements, laboratories, and formative simulation. Fellows also serve as coaches for their students, meeting one-on-one twice per semester. Students in the Late Clinical Experience (M3-M4) are dispersed across eight clinical campuses from southeast Michigan to the Upper Peninsula; the community campus becomes their primary structure for learning and support.

Curricular content is delivered using symptom-based presentations (e.g., chest pain). The core knowledge and skills for each topic are integrated within 96 C3 documents, freely available at justintime.com. The C3 documents include capstone cases – prototypical patient presentations that are used to develop patient cases for small group and formative simulation learning, as well as OSCEs. The Early Clinical Experience and Middle Clinical Experience use C3s to scaffold learning experiences by week. An example (Joint Pain) is summarized in Table 4.

**Table 4.** Sample early clinical experience week: Joint pain.

Activity	Content
Weekend Learning Module	Overview of the week’s content, to prepare for Large Group Activity Self-assessments Independent learning activities.
Large Group Activity	Normal histology Pathogenesis and presentation of osteo- and rheumatoid arthritis Management of chronic pain
Formative Simulation	Clinical evaluation of patients with: •Acute low back pain •Joint stiffness and fatigue •Chronic knee pain Writing a health record Table-top simulation of joint painUltrasound to demonstrate knee anatomy
Post Clinic Group 1	Case-based presentations with discussion of relevant anatomy, histology and cell biology, physiology, pathology, and pharmacology
Post Clinic Group 2	Clinical presentation including discussion of anatomy, histology and cell biology, biochemistry, and pathology
Integrative Biomedical Lab	Focus on cartilage, bone, joint histology and pathology
Gross Anatomy Lab	Focus on autonomic nervous system and pain
Weekly Formative Assessment	Self-assessment questions covering the current week’s content and sampling prior week’s content

Formative simulation lays the foundation for students’ meaningful clinical work throughout the first 2 years of the curriculum. They receive intensive training in basic physical examination maneuvers (e.g., blood pressure measurement, cardiac auscultation) and office-based tests (e.g., urine dipsticks) before being placed in clinics. Thereafter, students have weekly simulation sessions linked to C3 topics. In the Middle Clinical Experience, they have additional sessions related to rotational learning objectives (e.g., pelvic examinations for women’s health).

Class time, such as scholar groups and Large Group Activities, is designed as flipped classrooms to enhance student engagement and self-directed learning [9]. Students are provided learning objectives and suggested reading material as part of the introductory Weekend Learning Module available at justintime.com and for specific sessions. Classroom activities require students to apply the information they have learned to patient cases, quizzes, and other discussions.

Students are placed in authentic clinical settings beginning in week 9 of the first semester. In the Early Clinical Experience segment, they work side by side with medical assistants and nurses in primary care clinics for 1–2 half days per week. Their experiences also serve as the foundation for a quality improvement or scholarly activity that they present to their peers and the faculty. In the Middle Clinical Experience, they complete 2–4-week medical and interprofessional rotations: Adult Wards, Case Management, Emergency Medicine, Newborn Nursery, Nursing, Nutrition, Palliative Care, Pediatric Wards, Pharmacy, Physical Therapy, Respiratory, and Women’s Health. Student goals for interprofessional rotations are to explore team member roles and systems of care, witness patient presentations from a new perspective, and link foundational scientific knowledge to clinical care. In the Late Clinical Experience, students progress through parallel courses: departmental clerkships and electives, on one hand, and longitudinal integrative courses known as Advanced Skills and Knowledge.

Assessment is scaffolded by the college’s competencies: Service, Care of Patients, Rationality, Integration, Professionalism, and Transformation (SCRIPT). Each competency has three or more components (Table 2), and each component has been elaborated into milestones that are used for course objectives during the curriculum’s integrative courses [8]. Each semester, the learning objectives are linked to assessment types, including multiple choice examinations (i.e., the Comprehensive Necessary Science Examination); OSCEs (i.e., the Progress Clinical Skills Examination); direct observations of clinical skills using either checklists or an entrustment scale; multisource feedback from preceptors, peers, interprofessional team members, and self; and portfolio items [8]. Assessment data is aggregated in a cloud-based dashboard, JustinTime, for review by students and their coaches, and for the purpose of course grade decisions.

Grade recommendations for integrated courses come from a 54-member Student Competence Committee. Committee members, working in subgroups, review student data in light of course objectives and competency expectations to recommend grades of Pass, Conditional Pass, or No Pass. Grade decisions are finalized by course directors, who may ameliorate grade recommendations based on emerging evidence. In keeping with a competency model, all non-passing grades must be remediated. For most courses, this is accomplished during the normal course of activities in the following semester, thus mitigating the need for curricular extensions.

## Discussion

The goal of this paper was to provide a narrative overview of a new competency-based curriculum as an example for other schools undertaking this work. Key take-aways focus on engagement of stakeholders, faculty development for new roles, creating sustainable administrative systems, and managing complexity.

Curricular reform is time-consuming and requires the talents and insights of many people. Major change, such as integrated courses, can threaten the identities and autonomy of departments and faculty members alike. Disciplinary courses such as biochemistry and anatomy were dissolved, and content was dispersed across five semesters of the Early and Middle Clinical Experiences. Some units felt disenfranchised. Instead of delivering lectures within or leading a dedicated course, individuals were asked to serve in ways without direct student content (e.g., generating content, grading assignments), or that pushed them beyond their personal fields of expertise and required longitudinal commitments (e.g., becoming a learning society fellow). Incorporating more basic and social scientists in the CDG visioning process may have abrogated this problem.

Scholar groups and formative simulation are teaching-intensive activities and require clinician-educators with sound foundations in pedagogy and the ability to precept outside their areas of expertise. CHM is a community-based medical school with a small cadre of employed clinician-educators, focused primarily on one of the two 4-year campuses. To cover the new teaching responsibilities, the college needed to contract with local health systems for clinician time and hire other faculty members on a part-time basis. From year to year, fellows have been pulled from their Shared Discovery Curriculum roles to lead other programs or meet the clinical demands of their practices. Departures have disrupted some of the desired continuity with students and created need for ‘on-boarding.’ Ongoing professional development has been critical for new and old members alike. The overarching academy structure of the learning societies has developed a weekly half-day session that addresses pedagogy, content knowledge, coaching and assessment skills, and scholarly inquiry.

The curriculum has many moving parts and complicated student schedules. No commercially available programs have met our needs to support scheduling, content delivery, or assessment needs. In some cases, we have been able to pull together a mix of third-party systems to support interactive large group activities or progress testing. For others, such as content delivery, collection of real-time workplace-based assessments, and student dashboards, we have developed our own systems such as JustInTime. Workarounds have created opportunities for error and inefficiencies that we still strive to address.

It was challenging to recruit and maintain clinical site for students in the Early and Middle Clinical Experiences. The new curriculum more than doubled the demand for clinical placements, straining local resources where there was already competition from other learner groups such as pharmacy, nursing, physician assistant, and even other medical students. For Early Clinical Experience students, we reached out to county health departments, private groups, and the medical groups for affiliated hospital systems. We looked beyond the greater metropolitan areas of our two 4-year campuses to nearby cities and towns, capping commute time from campus to 45 minutes each way. The Middle Clinical Experience posed greater difficulties as rotations such as emergency medicine, pediatric wards, adult wards, and newborn nursery required settings already used for CHM students in the Late Clinical Experience. To accommodate the changes and account for the meaningful patient contact students received as M2s, clerkship lengths were shortened and, in some cases such as pediatrics, shifted to ambulatory sites. We also partnered with smaller, critical access hospitals in nearby communities that had not had students in the past. We hired additional staff to aid in recruiting, developing, and maintaining relationships with the clinical sites.

In conclusion, creating and implementing the Shared Discovery Curriculum has been a complicated, time-consuming, and ultimately fulfilling endeavor.
